# Gene delivery into mouse retinal ganglion cells by in utero electroporation

**DOI:** 10.1186/1471-213X-7-103

**Published:** 2007-09-17

**Authors:** Cristina Garcia-Frigola, Maria Isabel Carreres, Celia Vegar, Eloisa Herrera

**Affiliations:** 1 Instituto de Neurociencias de Alicante. Universidad Miguel Hernández-CSIC, Campus de San Juan, Apt 18, San Juan de Alicante, Alicante 03550, Spain

## Abstract

**Background:**

The neural retina is a highly structured tissue of the central nervous system that is formed by seven different cell types that are arranged in layers. Despite much effort, the genetic mechanisms that underlie retinal development are still poorly understood. In recent years, large-scale genomic analyses have identified candidate genes that may play a role in retinal neurogenesis, axon guidance and other key processes during the development of the visual system. Thus, new and rapid techniques are now required to carry out high-throughput analyses of all these candidate genes in mammals. Gene delivery techniques have been described to express exogenous proteins in the retina of newborn mice but these approaches do not efficiently introduce genes into the only retinal cell type that transmits visual information to the brain, the retinal ganglion cells (RGCs).

**Results:**

Here we show that RGCs can be targeted for gene expression by in utero electroporation of the eye of mouse embryos. Accordingly, using this technique we have monitored the morphology of electroporated RGCs expressing reporter genes at different developmental stages, as well as their projection to higher visual targets.

**Conclusion:**

Our method to deliver ectopic genes into mouse embryonic retinas enables us to follow the course of the entire retinofugal pathway by visualizing RGC bodies and axons. Thus, this technique will permit to perform functional studies *in vivo *focusing on neurogenesis, axon guidance, axon projection patterning or neural connectivity in mammals.

## Background

Cells in the retina are packaged into highly ordered anatomical layers each with a specialized function. During eye development, the cells in the ventricular zone undergo successive periods of proliferation and differentiation to generate seven types of retinal cells in a precise order (six types of neurons and one glial cell type). Neurogenesis occurs in the mouse retina between E13 and P10, with exit from the cell cycle reaching a maximum at E18-P0 [1]. Postmitotic retinal cells are generated in an highly ordered fashion from the pool of cycling progenitors, with ganglion, amacrine and horizontal cells originating first and rods, bipolar and Müller glia cells differentiating last.

Among the different cell types in the retina, ganglion cells (RGCs) are the only ones whose axons leave the retina, transmitting visual information to the brain. Once RGCs differentiate, their axons exit the eye through the optic disc in the centre of the retina, and they bundle together to form the optic nerve. Mouse RGC axons grow to the ventral diencephalon and while the majority of them cross the midline and form the optic chiasm, a small proportion of them do not. After sorting in the chiasm, the axons extend dorsally through the optic tracts to reach their principal synaptic targets, the superior colliculi (SC) and the lateral geniculate nucleus (LGN). Visual projections to higher brain targets are organized such that two adjacent RGCs in the retina are connected to two adjacent points in the target field. This topographic arrangement allows a continuous image of the visual field to be projected onto the surface of the target structure.

Gene expression profiles in developing retinal tissue during neurogenesis have been defined recently [2] and together with efforts to identify genes involved in axon guidance, axon targeting or neural connectivity in the retina, these studies have identified a number of candidate genes that may participate in the development of the visual system. However, functional studies must be performed to fully investigate the specific role of each candidate gene. Gene targeting or transgenesis-based strategies are time-consuming, arduous and expensive procedures, and they require the use of specific promoters that are not always available.

In this article we describe a method to ectopically express genes in RGCs during early stages of embryonic development. Using this approach it is possible to visualize the shape and location of RGCs at different stages of retinal differentiation. Moreover, the projection of RGCs expressing ectopic genes can be monitored along the retinofugal pathway, and even at the optic chiasm, LGN and SC.

## Results

### Gene delivery into the embryonic retina

Previous studies have demonstrated the versatility of electroporation as a method to deliver DNA into the developing telencephalic vesicles [3-6], as well as into certain retinal cell types in newborn mice [7]. However, gene delivery into the embryonic mammalian retina has not yet been achieved. We have adapted a method for in utero electroporation of cortical neurons [8], to deliver genes into the mouse retina during development, and more specifically into RGCs and amacrine cells.

Pregnant females were anesthetized and attached with adhesive tape to the cover of a 150 mm plastic Petri-dish by the anterior and posterior limbs. This immobilization facilitated their rotation and improved the injection of the DNA into the eyes of the embryos. The abdominal cavity was then opened and the uterine horns were exposed. Using a graduated pulled glass mouth-micropippette and with the aid of by a portable stereoscope, green fluorescent protein (GFP)-expressing plasmids were injected monocularly through the uterus wall into each embryo. We found it more convenient to use a graduated mouth-micropipette than a picospritzer because it facilitated the rotation of the mother when access to the embryo's eye was restricted, making it difficult to inject the DNA. However, a picospritzer could also be used in cases where very precise volumes must be injected. In the plasmids used, GFP was driven by the CAG promoter (chicken β-actin promoter with CMV enhancer), a very strong and ubiquitous promoter that is similar to those described previously [6,7]. We also used the cytomegalovirus (CMV) promoter but the levels of GFP expression were lower than those achieved with the CAG promoter.

**Figure 1:**
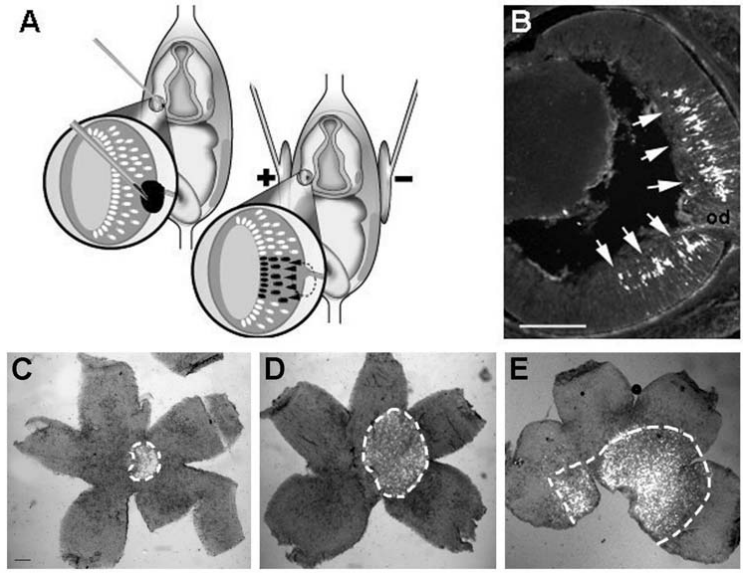
**Gene targeting into the embryonic retina**. (A) Schematic representation of retinal electroporation in utero. A small amount of DNA is injected into the embryo's eye through the uterine wall (left), and then electric pulses are passed using paddle electrodes. The result is the delivery of DNA to a subset of retinal cells (right). Only when the positive electrode was located on the injected eye was the electroporation successful. (B) Retinal section of an E16 embryo electroporated at E13. GFP expressing cells can be detected in the central part of the retina (arrows), surrounding the optic disc; scale bar: 200 μm. (C, D, E) Flattened whole mounts of E16 retinas electroporated at E13 after injection of different volumes of GFP-plasmid solution (0.2 μl, 0.5 μl and 1 μl respectively of a 1 μg/μl DNA solution) show the increase in the number cells targeted in the central retina (cells in the dashed circle). Scale bar: 500 μm.

Once the plasmid had been injected, an electric current was then passed through the head of each embryo using tweezer-type electrodes (Figure 1A), all embryos in each litter being electroporated. In one litter, the anode was positioned on the uninjected eye and the cathode on the injected eye while in a second litter, we examined the effects of applying the electrical current in the opposite direction. Embryos were sacrificed at different times and most of them had a normal appearance. GFP expression was only detected in retinal cells of embryos electroporated with the positive electrode on the injected eye, indicating that the DNA is transduced from the scleral side where undifferentiated and newly post-mitotic cells are located (Figure 1A). The procedure did not appear to be particularly harmful as approximately 90% of the injected embryos survived the electroparation. Moreover, about 70% of them expressed GFP in the retina and no damage was evident in the electroporated retinas (Figure 1B).

As expected, the number of GFP expressing cells in the retina varied according to the volume of DNA solution injected. Smaller injection volumes covered a smaller area of the retina and thus, the DNA was accessible to fewer cells. To confirm this hypothesis, electroporations were performed in the retina using different concentrations of DNA in the same volume of (0.2 μg/μl, 0.5 μg/μl, 1 μg/μl and 2 μg/μl). At 0.2 μg/μl no GFP expression was detected, while at the remaining concentrations examined no differences were observed in the area expressing GFP (Data not shown).

### Time course of expression in electroporated cells

**Figure 2:**
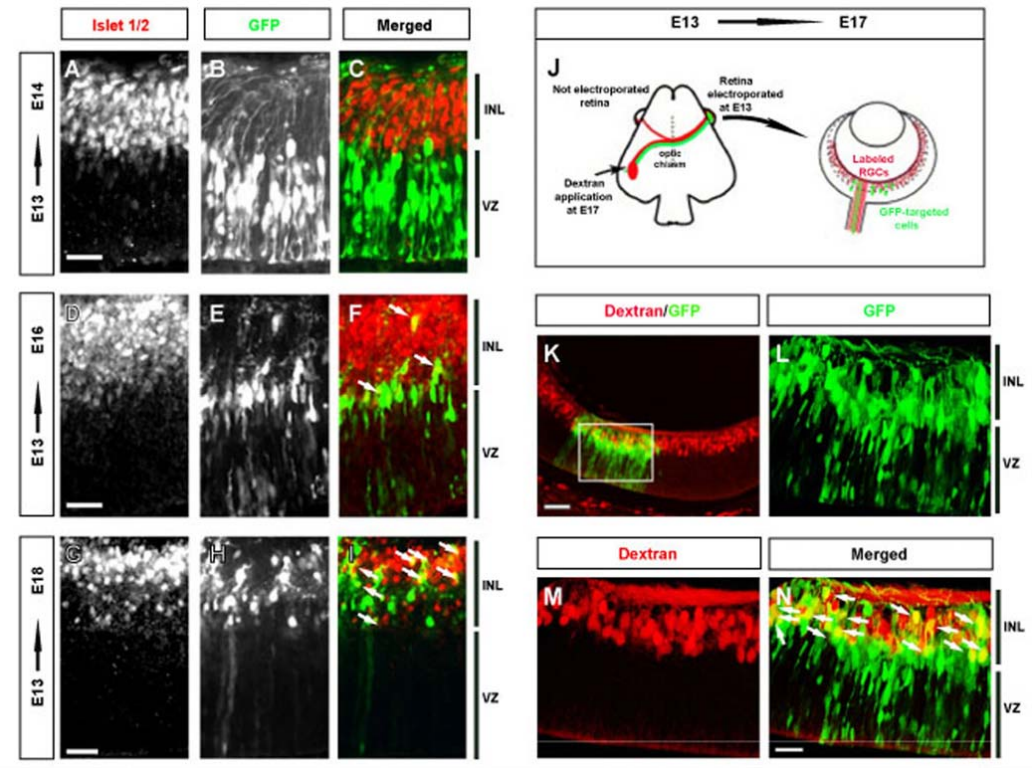
**Visualization of the development of the retinas targeted at E13**. (A-I) Retinas from E13 embryos were electroporated with GFP-bearing plasmids and sacrificed at E14, E16 or E18. Left panels show retinal sections from electroporated embryos incubated with anti-Islet1/2 antibodies to detect post-mitotic RGCs. Middle panels show targeted cells in the same retinal sections. Note that axons projecting to the inner layer can already be visualized in panel B at E14. Right panels show the merged images. At E16 GFP-positive cells are located closer to the inner layer (labelled by Islet 1/2, red) and a few double-labelled cells are observed (white arrows). At E18 the majority of the electroporated cells are located in the inner retinal layer and many of them are positive for Islet1/2. Scale bar: 20 μm. (J) Diagram showing the retrograde labelling paradigm. Dextran-rhodamine is applied at E17 in the optic tract (red) contralateral to the retina that was electroporated at E13 (green). The typical distribution of dextran-labelled cells and axons in the contralateral retina at E17 are shown (red), together with the GFP targeted cells that were electroporated at E13. (K) Retinal section electroporated at E13 (green cells) and retrogradely labelled with dextran-rhodamine (red cells). In all of the merged images, the double/labelled cells are yellow and they are indicated by white arrows. Scale bar: 100 μm (L-N) High magnification of the boxed area in K. Scale bar: 25 μm INL, Inner layer; VZ, ventricular zone.

To determine the identity of the electroporated cells, embryonic retinas were analysed at different time points after their electroporation at E13. One day after electroporation, at E14, GFP expression could already be observed in many cells in the ventricular zone (VZ) of the neural retina. However, by E16 most of the positive cells were located in a more internal layer than at E14, and by E18 almost all GFP positive cells were positioned in the innermost retinal layers where post-mitotic amacrine and RGCs are located (Figure 2).

**Figure 5:**
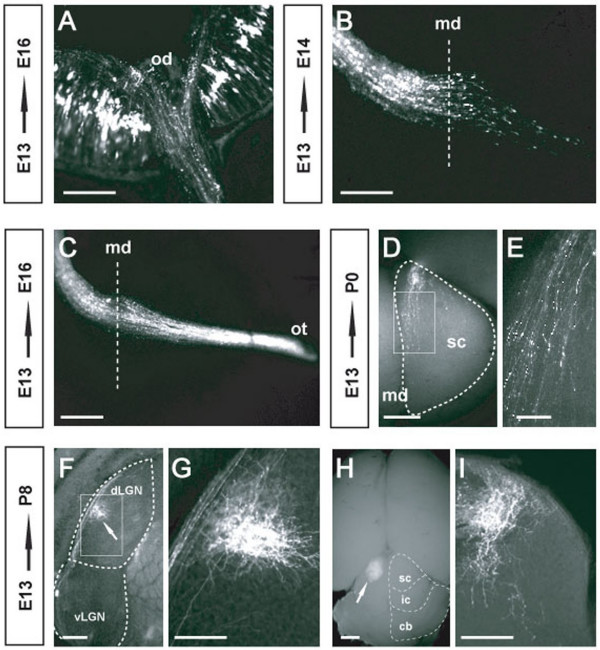
**The entire retinofugal pathway can be visualized when RGCs are targeted**. (A) GFP-expressing axons exiting the retina through the optic disc. (B) 24 h after electroporation many growth cones from targeted RGCs are observed at the optic chiasm. (C) Retinal axons are seen in the optic tract three days after electroporation. (D) In newborn animals electroporated at E13, individual retinal axons expressing GFP project into the superior colliculus. (E) Higher magnification of (D) showing individual axons within the superior colliculus. (F) The location of the axons from targeted cells can be detected in the LGN of frontal brain sections of P8 animals after electroporation at E13. (G) Higher magnification of (F) shows the precise location of individual axons. (H) RGC axons electroporated at E13 in the retina terminate in the superior colliculus at P8 (arrow). (I) A frontal section through the superior colliculus of the same animal shown in (H). Od, optic disc; on, optic nerve; md, midline; ot, optic tract; sc, superior colliculus; dLGN, dorsal lateral geniculate nucleus; vLGN, ventral lateral geniculate nucleus; ic, inferior colliculus; cb, cerebellum. Scale bars: 100 μm in E; 200 μm in A, B, C, F, G, I and 500 μm in D, H.

To determine the proportion of GFP positive cells that were in fact RGCs, retinal sections were labelled with the Islet1/2 antibody. Although the relationship between the onset of Islet1/2 expression and axonogenesis is unknown, this transcription factor is a known marker of RGCs during development [9]. Islet1/2 recognized cells in the most internal layer of the retina, which constitutes the final destination of RGCs during development. On E14, 24 hours after electroporation, GFP-expressing cells displayed a complementary pattern to that of the RGCs labelled with Islet1/2, and no double labelled cells were found (Figure 2A,B,C). However, 72 hours after electroporation GFP-expressing cells were located in a layer containing differentiated RGCs and a few double-labelled cells could be detected (Figure 2D,E,F). Despite the lack of Islet1/2 staining at E16 in the electroporated cells, many axons exiting the retina were visualized at this stage (Figure 2B, see also Figure 5A). Hence, at least some of the electroporated cells appeared to be RGCs.

To test whether we were targeting RGCs, we labelled RGCs retrogradely in embryos that were electroporated at E13 by applying axonal tracers to the optic tract contralateral to the injected eye (Figure 2J). In retinal sections from these animals, several GFP positive cells were filled with dextran indicating that they were indeed RGCs (Figure 2K–O). Interestingly, most of the double-labelled cells were not located in the most internal retinal layer, the future RGC layer. These results are in agreement with previous observations in the ferret retina suggesting that axons may be generated before the parental cell bodies have migrated to their final position [10].

### The cells electroporated at E13 form amacrine cells and RGCs

**Figure 3:**
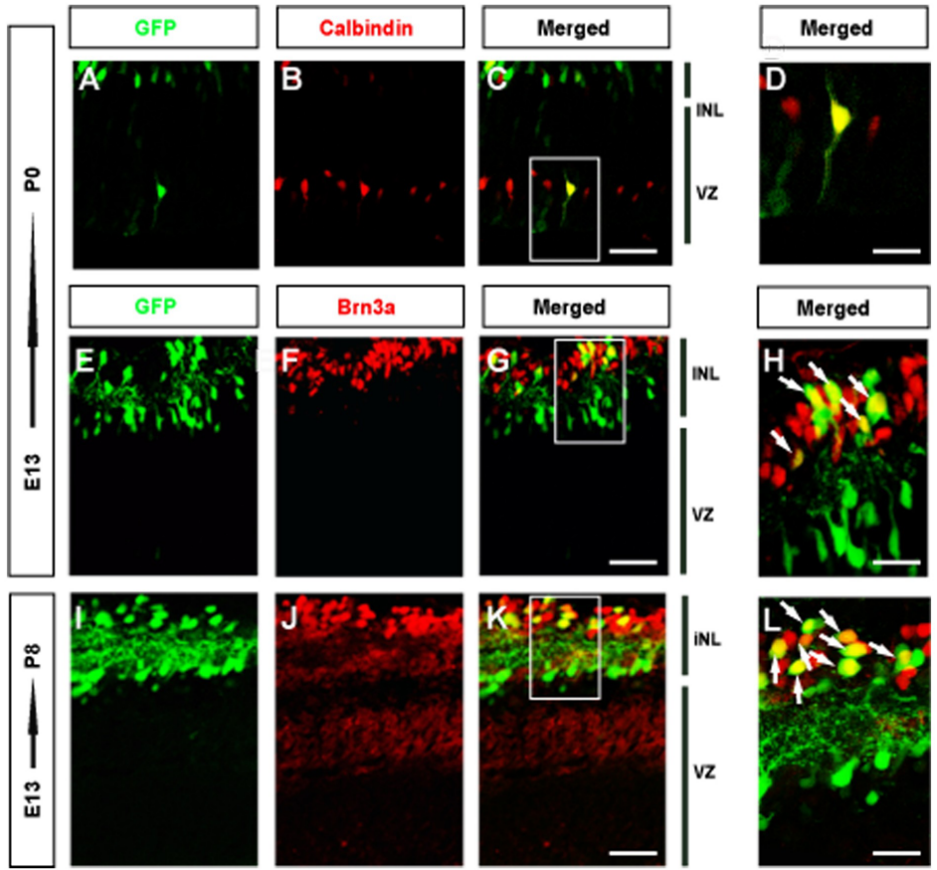
**Visualization of postnatal retinas electroporated at E13**. Retinas from E13 embryos were electroporated with GFP-bearing plasmids and sacrificed at P0 or P8. Retinal sections from electroporated embryos were incubated with anti-Calbindin (A-D) or anti-Brn3a (E-L) antibodies to identify horizontal cells and post-mitotic RGCs, respectively. (A-C) Calbindin staining on electroporated retinal sections at P0. (A) Shows the electroporated cell population at P0. Note that the vast majority of electroporated cells are distributed between the RGC and INL retinal layers but also, infrequent GFP labelled cells can be observed in the VZ. (B) Calbindin staining performed on electroporated retinal sections (C) Co-localization of calbindin and GFP (yellow cells) in a single cell located deep in the ventricular zone. A few amacrine cells are also positive for calbindin in the INL. Scale bar: 50 μm. (D) Higher magnification of a single cell in the ventricular zone that was electroporated at E13 and stained for calbindin at P0 indicating that it is a horizontal cell. Scale bar: 25 μm. (E-G) Sections of P0 retinas that were electroporated at E13, and stained for Brn3a. Scale bar: 50 μm. (I-K) Staining of electroporated retinal sections with the anti-Brn3a antibody at P8 when RGCs have reached their final location at the retinal surface. Note that the majority of GFP expressing cells located at the RGC layer co-localize with Brn3a (yellow cells), indicating that they are RGCs. Scale bar: 100 μm. High-magnification of GFP-expressing RGCs double-labelled with anti-Brn3a at P0 (H) and P8 (L). Scale bar: 25 μm RGC, retinal ganglion cell layer; INL, internal nuclear layer; VZ, ventricular zone.

When retinas electroporated at E13 were evaluated postnatally, almost all GFP-expressing cells were positioned in the superficial layers of the inner retina, which displayed the typical disposition of the RGCs, and the inner superficial layers were separated by the inner fibre plexus. Interestingly, sporadic GFP expressing cells were also found deep in the ventricular zone (Figure 3). We quantified the relative contribution of GFP expressing cells to each layer in P8 retinas, a stage at which RGC genesis has principally concluded. The majority of electroporated cells (99.25% ± 0.417) were found in the superficial layers of the retina while the remainder (0.75% ± 0.42) belong to the deeper portion of the ventricular zone. GFP positive cells in the inner layers contribute almost equally to the retinal ganglion cell layer (47.25% ± 0.84) and to the inner nuclear layer (52% ± 1.27, Figure 3I).

To determine the final identity of electroporated cells in newborn and P8 retinas we examined the expression of the transcription factor Brn3a, a marker of a large population of RGCs that appear late in development (Figure 3E–K). Accordingly, 70% of electroporated cells located in the RGC layer expressed Brn3a in P8 retinas and hence, corresponded to retinal ganglion cells (Figure 3H,L). Staining for calbindin, a marker for horizontal cells, in newborn retinas also revealed that the few GFP expressing cells located in the ventricular zone were horizontal cells (Figure 3A–D). We cannot rule out the possibility that other retinal cell types were transfected in low numbers when we electroporated at E13 but in our experiments, we failed to detect any other retinal cell type.

According to their final location in the INL and given their appearance, we assume that the rest of electroporated cells belonged to the diverse population of amacrine cells. Indeed, electroporated cells in the RGC layer that did not express Brn3a may either correspond to displaced amacrine cells or to a population of RGCs that do not express Brn3a.

In summary, electroporation at E13 mainly transfects retinal ganglion cells and amacrine cells, as well as a few sporadic horizontal cells. These findings are in accordance with the proposed competence model of retinal neurogenesis in which these cell types are generated simultaneously in the developing retina [1,11].

### The timing of electroporation affects the area of gene expression in the retina

**Figure 4:**
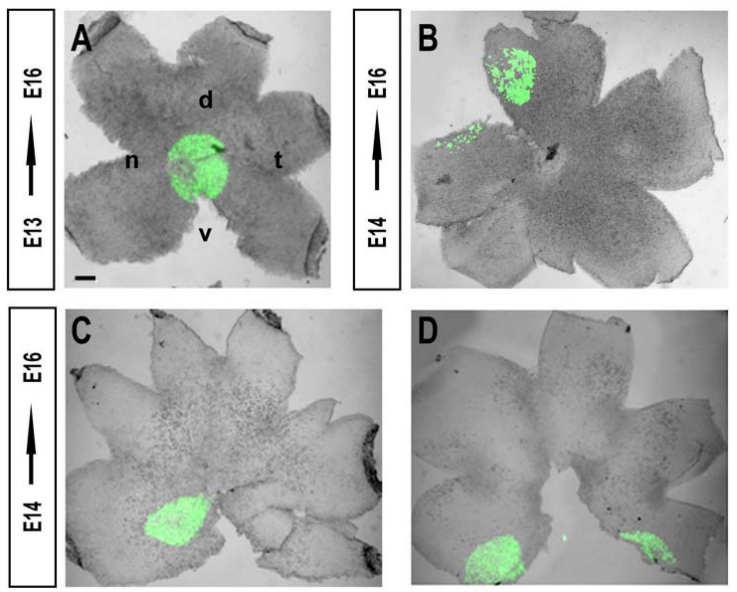
**The timing of electroporation affects the area of gene targeting**. (A) In flattened whole mount E16 retinas electroporated with GFP-plasmids at E13, the cells in the central retina are targeted (green cells). In contrast, when electroporation is performed at E14 the location of GFP-expressing cells varies depending on the position of the electrodes. Examples of retinas electroporated at E14 in the medial dorso-nasal region (B) medial ventronasal (C) or the very peripheral ventronasal retina (D). d, dorsal; n, nasal; t, temporal; v, ventral. Scale bar: 500 μm.

Since not all the cells in each retina expressed GFP after in utero electroporation, we wondered whether some regions of the retina took up the plasmid preferentially. In retinas electroporated at E13 and examined at E16, GFP-expressing cells were always located in the central retina surrounding the optic disc (Figure 4A). However, when retinas were electroporated one day later at E14, GFP expression could be detected in various regions of the retina depending on the position of the electrodes. Indeed, GFP expressing cells could be found in the dorsal-intermedial retina (Figure 4B), the ventral-intermedial retina (Figure 3C), in the peripheral retina (Figure 4D), or in any other area of the retina (data not shown).

The differences observed in the location of the electroporated cells between E13 and E14 probably reflects the increase in the size of the retina. At E13, the retina is sufficiently small that the DNA injected extends over the entire retina. As a consequence, most of the electroporation-competent cells incorporated the plasmid. Nevertheless, at E16 peripheral cells in retinas electroporated at E13 did not express GFP because newly generated cells had been added to the edges of the retina during this period. In contrast, at E14 the retina was larger and the plasmid DNA injected was accessible to a proportionally smaller area of the retina. Thus, the location of electroporated cells at E14 is primarily driven by the position of the electrodes.

### Electroporation of RGCs enables the entire retinofugal pathway to be visualized

The first RGC axons enter the optic nerve around E12 and pass throughout the optic chiasm between E13–E16. At the optic chiasm, retinal fibres diverge and project into the ipsilateral or contralateral side of the brain. After the sorting of retinal axons at the optic chiasm, RGC axons then establish topographic maps in the LGN and SC between E18 and P8. Because RGC axons project over long distances to reach the LGN and SC, we wondered whether we could follow the full length of the retinofugal pathway by visualizing GFP incorporated into electroporated RGCs. GFP-expressing axons could be seen to exit the retina through the optic disc (Figure 5A) and in embryos electropored at E13, growth cones could be seen to cross the midline as soon as 24 hours post-electroporation (Figure 5B). Three days after electroporation, the majority of the GFP-expressing axons had already crossed the midline chiasm and entered the brain through the optic tract (Figure 5C). One week later, at P0, many green axons could be visualized at the level of the LGN and in the SC (Data not shown and Figure 5D, E). Finally, at P8 when the projection patterns of axons had been established, GFP-labelled synaptic terminals were evident in the LGN and SC (Figure 5F,G,H,I).

## Discussion

The retina is an accessible component of the CNS that has a well-defined cytoarchitecture. Accordingly, it is a structure in which neurogenesis, cell fate specification and the signals that are responsible for promoting cell survival versus programmed cell death during development can be studied in detail. Within the retina, RGCs are the only neurons that send visual information to higher brain areas and their axons project to the midbrain targets, the SC and the LGN. Through numerous studies, molecular signals that direct the topographic information for the precise wiring during development have been identified. Indeed, transcription factors, guidance molecules, extracellular matrix proteins, neurotrophic factors, and cell death regulating factors are known to be involved in the formation of a precise retino-thalamic and retino-collicular map.

Many studies to identify factors that control the development of the visual system have been carried out in the chick, zebrafish and *Xenopus*. These are animal models in which their extra-uterine development makes it easy to manipulate the embryonic retina. These studies have provided fundamental information on how retinal neurogenesis and topographic retinofugal projections form during embryogenesis. In mammals, time-consuming and complicated gene-targeting approaches are required to elucidate the function of specific molecules in retinal development or to confirm the results obtained in non-mammalian species [12-19]. Here, we have adapted a fast and relatively easy technique to transfer genes into the embryonic retina that will greatly facilitate a wide variety of studies on the development of the visual system in mammals.

The vertebrate neural retina is patterned along all its axes, and retinal differentiation initiates in the central retina, expanding to the periphery [20]. The position of each RGC along the anterior-posterior (A-P) and dorsal-ventral (D-V) axes (its positional identity) determines its connectivity and is crucial for the formation of the topographic maps. Our method for in utero retinal electroporation permits the ectopic expression of genes along the central-peripheral, dorso-ventral and anterior-posterior axes of the embryonic retina. Hence, this technique enables the position of the electroporated RGC cell bodies to be precisely matched with the location of their axonal projections in the LGN and SC.

Cell-fate determination and axonal navigation are achieved by the combinatorial activity of multiple transcription factors and axon guidance molecules [21]. The procedure described here may be used to promote the simultaneous expression of several genes in gain-of-function experiments, or to downregulate genes of interest by electroporation of specific iRNA's.

Studies of neurogenesis can also benefit from this in utero electroporation technique. The six types of retinal neurons are generated in a very orderly manner that is conserved across all species. Ganglion, amacrine and horizontal cells differentiate first, from E11 to P0 in the mouse, and bipolar cells, Müller glia and rods are produced last [11]. Electroporation in neonatal mice leads to the expression of ectopic genes mostly in rods and in a small number of bipolar and Müller glia cells [7]. While other authors have reported some electroporation of RGCs in the adult retina [22,23], we and others have failed to reproduce this result [7]. However, we have accomplished highly efficient gene delivery to RGCs during embryogenesis. One possible explanation for our results is that cycling cells are the more receptive to electroporation during development. During embryogenesis, all progenitor cells in the ventricular zone could be electroporated, while only those cells that exit the cell cycle shortly after electroporation (RGCs and amacrine cells) will take up sufficient plasmid to be visualized. Indeed, in targeted progenitors that continue cycling the plasmid incorporated will be diluted out over the divisions later and the levels of GFP would become too low to be detected. This hypothesis is supported by our results showing that ganglion, amacrine and a few horizontal cells are electroporated at E13, since all these cell types are generated simultaneously during development [1,11]. Although it has been suggested that a number of cone cells also differentiate during this period, a significant number of transfected cones was not detected in our experiments. One possible explanation for this is that the production of cones takes place in a very restricted time window, just before or after our electroporation experiments were performed. Indeed, although birth dating of most retinal types has been investigated intensely, cone differentiation is poorly understood. It is significant that in a recent study, GFP labeled RGC or horizontal cells were not detected when the retina of newborn mice was electroporated, but they did detect GFP labeled cone cells (Matsuda and Cepko, 2004). Thus, perhaps the time at which cones are born does not adhere strictly to that described in much earlier studies [24].

The fact that Islet1/2 positive cells do not express GFP 24 hours post-electroporation once again suggests that post-mitotic cells are not targeted. In addition, this theory is also consistent with studies demonstrating the electroporation of RGCs in P1 ferrets [25], particularly because in this organism the peak of RGC generation occurs between P3 and P6 [26].

## Conclusion

We have described here a technique for efficient gene delivery into RGCs by in utero electroporation. Using this approach, cells at different stages of retinal differentiation, as well as the entire retinofugal pathway, can be visualized by GFP fluorescence. The use of this technique will benefit research into different aspects of nervous system development including neurogenesis, axon guidance, patterning of axon projections, synaptogenesis, refinement of connections and visual processing.

## Methods

### Animals and DNA constructs

All the animals used in this study were pigmented C57BL6/J pregnant females, since pigmented eyes are easier targets for DNA injection through the uterus wall. Although CD1 albino embryos were also successfully electroporated, the use of pigmented mice increased the efficiency of the protocol. Animals were treated in accordance with Spanish and European Union guidelines for the Care and Use of Laboratory Animals.

A plasmid containing the GFP coding sequence under the control of the CAG promoter (pCAG-GFP) was used in all the electroporation experiments performed here, similar to that previously described [6,7]. Plasmid DNA was purified using a conventional midiprep kit (Qiagen, Valencia CA) and resuspended in TE. DNA was generally injected in a 1 μg/μl solution.

### In utero electroporation

Timed-pregnant C57BL6/J females were anesthetized by intraperitoneal delivery of sodium pentobarbital (0.625 mg per 10 g body weight) and Rytodrine (0.1 ml of a 14 mg/ml solution). The abdomen was opened and the uterine horns exposed. The DNA solution (0.2 μg/μl, 0.5 μg/μl, 1 μg/μl or 2 μg/μl + 0.03% fast green in PBS) was injected into one eye of each embryo using a graduated pulled-glass micropipette. The head of each embryo was placed between tweezer-type electrodes (CUY650-P5 Nepa GENE, Chiba, Japan) and five square electric pulses (38 V, 50 ms) were passed at 1 s intervals using an electroporator (CUY21E, Nepa GENE). The wall and skin of the abdominal cavity were sutured and closed, and the embryos were allowed to develop normally.

### Immunostaining of retinal sections and visualization of the retinofugal pathway

Electroporated embryos or pups were perfused and fixed in 4% paraformaldehyde (PFA) for 1–3 days. The heads were cryopreserved in 30% sucrose in PBS and then embedded in Cryoblock (Labonord). Cryosections (30 μm) were obtained and non-specific binding was blocked in 5% Normal goat serum (NGS) in PBS + 0.2% Tween-20 for 1 h at room temperature. The sections were incubated overnight with the anti-Islet1/2 antibody (diluted 1:10000, a generous gift of Dr. Tom Jessell and Susan Morton, Columbia University), the anti-Calbindin antibody (1:2000, Swant), or anti Brn3a (1:200, Chemicon) diluted in 1% NGS-PBS-0.2% Tween-20 at 4°C. A Cy3-conjugated rat anti-guinea pig (Jackson Immunoresearch), Alexa-Fluor-546-goat anti-rabbit (Molecular Probes) and Alexa-Fluor 546-rabbit anti-mouse were used as secondary antibodies, respectively.

To visualize retinal projections at different levels of the retinofugal pathway, the brains were fixed in 4% PFA, embedded in 3% agarose and sectioned in a vibratome (100 μm). The hypothalamus, LGN and SC areas were photographed by conventional microscopy.

### Quantification of P8 retinas

Retinal sections from mice were analyzed (n = 2) and a total number of 398 cells were included in the study. Each given percentage was calculated as the average percentage ± SEM

### Dextran retrograde labelling of RGC axons

Embryos were electroporated with CAG-GFP at E13, sacrificed at E17, and the embryo's heads were dissected out, the palate removed and the optic chiasm exposed. Dextran tetramethyl rhodamine (MW 6000, Molecular probes) was applied to the contralateral optic tract for retrograde transport to the retina. Heads were maintained in carbonated ACSF buffer for 4 hours at RT, in cold ACSF overnight and fixed in 4% paraformaldehyde the next morning. Retinal sections (100 μm) were obtained using a vibratome

## Competing interests

The author(s) declares that there are no competing interests.

## Authors' contributions

CG-F optimized and carried out the electroporation experiments, as well as performing the statistical analysis and participating in the design of the study; MIC. assisted CG-F with the electroporations and immunohistochemistry; CV maintained the mice colony and participated in the immunohistochemical assays; EH conceived the study, carried out the data analysis and drafted the manuscript. All authors read and approved the final version of the manuscript.
